# Simple Serum Pancreatic Ductal Adenocarcinoma (PDAC) Protein Biomarkers—Is There Anything in Sight?

**DOI:** 10.3390/jcm10225463

**Published:** 2021-11-22

**Authors:** Monika Kapszewicz, Ewa Małecka-Wojciesko

**Affiliations:** Department of Digestive Tract Diseases, Medical University of Lodz, Kopcinskiego 22, 90-153 Lodz, Poland; ewa.malecka-panas@umed.lodz.pl

**Keywords:** PDAC biomarkers, early diagnostic, ELISA

## Abstract

A poor PDAC prognosis is due to a lack of effective treatment and late diagnosis. The early detection of PDAC could significantly decrease mortality and save lives. Idealbiomarkers for PDAC should be cost-effective, detectable in easily accessible biological material, and present in sufficient concentration in the earliest possible phase of the disease. This review addresses newly selected, simple protein biomarkers—new ones such as thrombospondin-2, insulin-linked binding protein 2, lysophosphatidic acid, and autotaxin and conventional ones such as Ca19-9, inflammatory factors, and coagulation factors. Their possible use in the early detection of PDAC, differentiation from benign diseases, prognosis, and treatment response prediction is discussed. We also address the usefulness of possible combinations of biomarkers in diagnostic panels.

## 1. Introduction

Pancreatic ductal adenocarcinoma (PDAC) is a highly aggressive malignancy that is estimated to become the most deadly among all gastrointestinal cancers. It ranks fourth in terms of mortality in western countries [1,2].

Despite advances in the treatment and understanding of the molecular mechanisms of carcinogenesis, less than 5% of patients survive 5 years after initial diagnosis. Most patients with PDAC (80–85%) are diagnosed with the locally advanced or distant metastases stage when radical surgical treatment is no longer possible [2,3].

PanINs, intraductal papillary mucinous neoplasms (IPMNs), and mucinous cystic neoplasms (MCNs) are considered precursor lesions of PDAC. PanINs are microscopic noninvasive flat or papillary lesions that develop in small pancreatic ducts. 

There are four grades of PanINs depending on architectural and cytological atypia in pancreatic ducts: the lowest grade (PanIN 1A and PanIN 1B), intermediate grade (PanIN 2), and high grade (PanIN 3), which refers to carcinoma in situ. The early curable PDAC grades do not cause any symptoms and are not visible in routine imaging techniques, such as endoscopic ultrasound (EUS), magnetic resonance (MR), and computer tomography (CT). Therefore, the early detection of pancreatic cancer is difficult and rarely happens [4].

**Figure 1:**
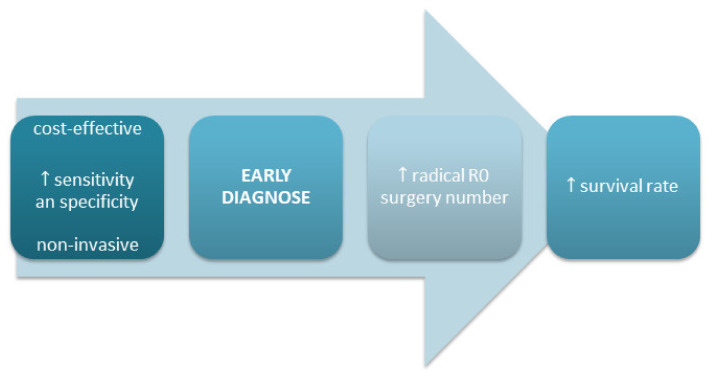
Features of ideal PDAC biomarkers [3].

The search for PDAC diagnostic, prognostic, and predictive markers with adequate sensitivity and specificity remains unsatisfactory. Diagnostic biomarkers should detect PDAC at an early and potentially curable stage. Prognostic markers provide information about the progression dynamics of the disease, and they can help to elaborate an adequate management strategy. Predictive markers are used to predict the efficacy of particular treatments (Figure 1) [3].

For the time being, only the Ca19-9 antigen has been recognized by the U.S. Food and Drug Administration (FDA) as a diagnostic marker, and it is widely used [5].

Most other investigated biomarkers are expensive or at an early stage of evaluation and therefore cannot be used in everyday clinical practice. An ideal biomarker for PDAC should exhibit high sensitivity and specificity, be non-invasive, and be cost-effective to improve early diagnosis and subsequent treatment (Figure 1).

Here, we present the latest trends in the search for simple, protein diagnostic, prognostic, and predictive PDAC biomarkers.

## 2. Diagnostic Biomarkers

### 2.1. Ca19-9

Ca19-9, a carbohydrate antigen, is a sialylated form of a Lewis group antigen present on the surface of erythrocytes and other blood cells [5]. 

The sensitivity and specificity of Ca19-9 in detecting PDAC in symptomatic patients are 79–81% and 82–90%, respectively [1].

Nevertheless, patients with a specific Lewis genotype (about 5–10% of the Caucasian population) do not express Ca19-9, which may give false negative results. It should be emphasized that elevated Ca19-9 levels may be associated with other pathologies, such as pancreatitis, liver cirrhosis, acute cholangitis, ascites, systemic lupus erythematosus, colorectal cancer, gastric cancer, and endometrial cancer [6].

Ca19-9 it is not useful in general population screening for early PDAC in healthy populations. In a study involving 70,940 asymptomatic individuals, PDAC was detected in only 4 patients from among 1063 individuals with elevated Ca19-9 marker levels [7].

On the other hand, the combination of Ca19-9 with other biomarkers in high-risk populations (such as those with diabetes mellitus, pancreatitis, PDAC family history and, smoking) improves its diagnostic performance. Many authors and guidelines indicate that in patients with high PDAC risk, such as those with hereditary pancreatitis, Lynch syndrome (HNPCC), familial adenomatous polyposis (FAP), Peutz-Jeghers syndrome, familial-atypical multiple mole melanoma (FAMMM), hereditary breast and ovarian cancer (HBOC), Fanconi anemia, Hippel-Lindau disease, Li-Fraumeni syndrome, ataxia telangiectasia, and a family history of PDAC (the occurrence of the pancreatic cancer in a minimum of two family members) should undergo an EUS examination together with serum Ca19-9 marker determination each year starting at the age of 35 [1,6,7].

In conclusion, Ca19-9 it is not useful in the early detection and screening of PDAC. The utility of Ca19-9 in the diagnosis of PDAC is limited due to its low sensitivity and specificity. Moreover, its Lewis antigen status should be investigated due to the possibility of false negative Ca19-9 results. Nevertheless, Ca19-9 may be helpful in the surveillance of patients at high risk of PDAC.

### 2.2. Peripheral Blood Monocyte (PBM)

Elevated PBM counts and peripheral blood monocytosis at diagnosis were observed in a study including 219 PDAC patients. PBM counts and temporal trends were analyzed over a 2-year pre-diagnostic period. The results showed that PDAC patients manifested monocytosis at diagnosis (23% vs. 8%; *p* < 0.001) and higher mean PBM count (x109/L) (0.73 vs. 0.59; *p* < 0.001) compared to controls. In the 24-month period before PDAC diagnosis, mean PBM counts were significantly higher in PDAC cases in the 6-month period before diagnosis compared to healthy subjects (0.69 vs. 0.61; *p* = 0.03). Additionally, PDAC patients with monocytosis at diagnosis demonstrated a decreased overall survival (OS) compared to those without monocytosis (1.9 months vs. 7.6 months; *p* = 0.001). Peripheral blood monocytosis were found to be a predictor of poor outcomes independent of tumor stage (*p* = 0.005) [8].

This simple and non-invasive laboratory test may be useful as a potential biomarker for the early detection of PDAC. Monocytosis is more frequent in PDAC patients at diagnosis compared to healthy subjects and mean PBM counts are significantly increased in PDAC cases at diagnosis and even 6 months before. These new findings might have a significant impact in the early diagnosis and clinical assessment of prognosis and treatment planning. There is a strong need to be verify and evaluate the role of elevated PBM values and monocytosis.

### 2.3. Thrombospondin 2 (THBS-2)

THBS-2 is a glycoprotein present in the extracellular matrix; by inhibiting tumor angiogenesis, it controls tumor growth and is considered to be one of the body’s defensive anti-cancer mechanisms [9].

The biological role of THBS-2 in angiogenesis and tumor growth was evaluated in an experimental study in transgenic mice. The mice were divided into two groups: the first one with human squamous carcinoma cell line A431 and the second one with an additionally injected THBS-2 overexpression vector. Cryostat sections of five THBS-2-expressing tumors were treated with the anti-CD31 monoclonal antibody. Blood vessels stained with CD31 were evaluated at three different magnifications in sections obtained from five tumors. The average vessel density, vessel size, and percentage of tissue area covered by vessels were determined with the computer-assisted analysis of representative digital images. The thinning of the small blood vessels was observed in tumors transfected with THBS-2 compared to control tumors. The vascular density was reduced by more than 50% in THBS-2-overexpressing squamous cell carcinomas compared to wild-type tumors, which demonstrated between 80 and 125 CD31-positive vessels per mm^2^ tumor area. Moreover, THBS-2-overexpressing tumors demonstrated reductions of the average vessel by more than 45%. The relative tumor area covered by vessels was reduced by 70% in THBS-2-expressing tumors compared to control tumors. Immunohistochemical analysis showed that the overexpression of THBS-2 in mice with carcinoma transplants was significantly correlated with tumor growth inhibition by more than 90% (*p* < 0.001); in the remaining group, the rapid growth of squamous cell carcinomas was observed, reaching a volume of 2000–3000 mm^3^ within 3 weeks. That results demonstrated that the tumor cell expression of THBS-2 potently inhibited the growth of human squamous cell carcinomas compared to tumors transfected with vectors alone. It was suggested that THBS-2 may suppress carcinogenesis, thus being an angiogenesis regulator [10].

The diagnostic role of THBS-2 was assessed in a study including various PDAC stages, benign pancreatic diseases, and healthy subjects. The THBS-2 level differentiated PDAC (*n* = 197) from healthy subjects (*n* = 140) with an AUC of 0.875 (95% CI = 0.85, 0.90). Furthermore, the AUC value for a combination of THBS-2 and Ca19-9 in differentiating PDAC from healthy control was 0.970 (95% CI = 0.96, 0.98). A combination panel of Ca19-9 and THBS-2 performed well across at the stages of resectable PDAC (stages I and II) with an AUC of 0.960 (95% Cl = 0.94, 0.98; *p* < 0.0001) and locally advanced and metastatic PDAC (stages III and IV) with an AUC of 0,980 (95% Cl = 0.97, 0.99; *p* = 0.0028). A Phase 2b study using 42 ng/mL and ≥55 IU/mL cut-off points for THBS-2 and Ca19-9, respectively, showed a specificity of 98% and a sensitivity of 87% in detecting PDAC in all PDAC cases compared to a healthy group. Furthermore, the combination of two markers allowed for the differentiation of PDAC from IPMN with an AUC of 0,952 and PDAC from chronic pancreatitis (CP) with an AUC of 0.867 compared to Ca19-9 alone [11]. A panel of THBS-2 and Ca19-9 may enable the early-stage detection of resectable PDAC and may be able to improve the prognosis. The combined expression THBS-2 and Ca19-9 showed a high accuracy in distinguishing PDAC from chronic pancreatitis. 

Another study confirmed the potential role of THBS-2 and Ca19-9 measured with serum ELISA in PDAC detection. A level of THBS-2 of over 66 ng/mL was significantly more frequent in the PDAC group (*n* = 82) compared to healthy subjects (24 ng/mL) (*n* = 50) with an AUC of 0.844 (95% CI: 0.784–0.904; *p* < 0.001). A level of Ca19-9 of over 312 IU/mL strongly differentiated PDAC from healthy subjects, which resulted in an AUC of 0.872 (95% CI: 0.817–0.927; *p* < 0.001). Furthermore, the expression of THBS-2 and Ca19-9 was examined in patients with resectable (stage I/II; *n* = 77) and locally advanced and metastatic PDAC (stage III/IV; *n* = 34). The optimal cut-off of THBS-2 was found to be 40.9 ng/mL. The combination of THBS-2 and Ca19-9 markers showed a high accuracy in diagnosing PDAC with a sensitivity of 90% and a specificity of 90%. Both markers together showed a similar performance for early- (AUC = 0.971) and advanced-stage (AUC = 0.911) PDAC [12].

The expression of THBS-2 could have potential as a diagnostic biomarker for patients with early or advanced PDAC. One of the advantages of THBS-2 is the ability to measure this marker in serum with an inexpensive ELISA test. There is a strong need for further validation studies that would define the best cut-off point for THBS-2 values to detect PDAC at an early stage. Moreover, a combined panel of Ca19-9 and THBS-2 may be additionally useful in the differentiation of patients with malignant and benign pancreatic diseases.

### 2.4. Insulin-Like Growth Factor Binding Protein 2 (IGFBP2), Insulin-Like Growth Factor 1 (IGF-1), IGFBP2/p65/EMT Axis

Recent diabetes detected after 50 years of age is strongly connected to PDAC development. The prevalence of carbohydrate-impaired tolerance and diabetes is as high as 40–80% in patients with this malignancy. Statistics show that on average 2 years before the diagnosis of PDAC, in the absence of visible tumor in imaging techniques, almost 75% of patients were diagnosed with diabetes [13].

It is currently proposed that proteins involved in diabetes development may provide a marker for early PDAC detection. Potential factors affecting pancreatic ductal proliferation pathways and apoptosis include hyperglycemia, hyperinsulinemia, and the increased expression of insulin-like growth factor 1 (IGF-1). In chronic pancreatitis (CP), pancreatic islet β-cell dysfunction results in reduced insulin and C-peptide levels. In contrast, high values of these hormones are observed in PDAC [14].

The insulin-like growth factor binding protein (IGFBP) complex consists of six polyproteins (IGFBP1-6) of similar structure that bind insulin growth factors (IGFs) and regulate their systemic half-life. Most (about 95%) of circulating IGF-1 molecules are bound by the IGFBP-3 protein. IGFBP2 blocks the binding of IGF-1 to its receptor and also functions independently of IGF-stimulating proliferation, the differentiation of normal or malignant cells, and the inhibition of anti-apoptosis [15]. There is extensive evidence that IGFBP2 is expressed in both plasma and tumors in numerous malignancies such as lung, prostate, and colorectal cancers [16].

Elevated IGBP2 levels were found to be useful in differentiating patients with early-stage invasive PDAC from controls with an AUC value of 0.706. Additionally, the authors-of this study demonstrated that a combination of IGBP2, IGFBP-3, and Ca19-9 significantly improved the accuracy of PDAC diagnosis with an AUC of 0.9 compared to Ca19-9 alone. Nevertheless, the IGBP-3 and Ca19-9 levels have also been found to be increased in benign pathologies, such as CP [16].

Insulin-like growth factor 1 (IGF-1) and insulin-like growth factor 2 binding protein (IGFBP2) are associated with an increased risk of cancer development, including pancreatic adenocarcinoma (PDAC). The IGF-1 and IGFBP2 proteins belong to the IGF axis, which plays a significant role in the development of disturbances in the course of PDAC [17].

The serum ELISA concentrations of IGF-1 and IGFBP2 in patients with newly diagnosed PDAC and healthy subjects were evaluated. A group consisting of 69 PDAC subjects had significantly lower serum IGF-1 levels compared to healthy subjects (45.83 ± 30.03 ng/mL vs. 70.66 ± 60.57 ng/mL, respectively; *p* < 0.0001). In contrast, the IGFBP2 levels of the PDAC group were significantly higher compared to the control group (225.06 ± 86.37 ng/mL vs. 51.92 ± 29.40 ng/mL, respectively; *p* < 0.0001). Moreover, the IGF-1 to IGFBP2 ratio of ≤0.85 detected PDAC with sensitivity of 100% and a specificity of 97%, so it could be a strong PDAC indicator [18].

Among 92 patients with PDAC diagnosed within the last 6 months and 83 subjects with CP, the serum IGFBP2 level was significantly higher in the CP subjects compared to the PDAC subjects (512.42 ± 299.77 ng/mL vs. 301.59 ± 190.36 ng/mL, respectively; *p* = 0.000082). Moreover, IGFBP2 levels were 9.9-fold increased in the CP patients (512.42 ± 299.77 ng/mL vs. 51.92 ± 29.40 ng/mL, respectively; *p* < 0.00001) and 5.8-fold increased in the PDAC patients compared to healthy controls (301.59 ± 190.36 ng/mL vs. 51.92 ± 29.40 ng/mL, respectively; *p* < 0.00001). Lower serum IGF-1 levels were observed in PDAC patients. The authors suggested that IGFBP2 may be a good biomarker in the diagnostics of these pancreatic diseases [19].

Recently, a study on pancreatic cancer cell lines showed that the elevated expression of IGFBP2 promoted the invasion and metastasis of PDAC cells by inducing NF-κB-dependent epithelial–mesenchymal transition (EMT). NF-κB inhibits apoptosis, the induction of proliferation, and the enhancement of angiogenesis processes, so it is considered to be an important factor in the processes of oncogenesis and PDAC progression. A significant correlation between serum IGFBP2 levels and OS in 80 patients with PDAC was shown. The patients whose tumors presented a high expression of IGFBP2 had shorter OS (13 months) compared to those whose tumor presented none or a low expression of IGFBP2 (20 months). Mouse models have been used to demonstrate how IGFBP2 promotes PDAC cell metastasis. In mice injected with IGFBP2-overexpressing cell lines, circulating IGFBP2 was elevated in primary and metastatic lesions compared to control line AsPc-1 cells. It was also observed that both lesions did not vary in size or weight, which indicates that IGFBP2 may be involved more in the invasion than proliferation process. Immunohistochemistry (IHC) staining in tumor sections from mouse models constructed by injecting mice with AsPc-1 cells transfected with IGFBP2-expression plasmids showed a positive correlation of IGFBP2 expression with nuclear p65 (*p* < 0.001) and vimentin (*p* < 0.001) expression and an inverse correlation with E-cadherin expression (*p* < 0.001). In a mechanistic model, the knockdown of IGFBP2 and p65 resulted in the decreased expression of nuclear protein p65 and vimentin and the increased expression of E-cadherin compared to levels in control cells. The Kaplan–Meier OS curves showed that high nuclear p65 and vimentin expression and low E-cadherin expression were associated with shorter survival [20]. It is known that the translocation of the p65 protein from the cytoplasm to the nucleus activates NF-κB. After its activation, the NF-κB/p65 complex is separated from the inhibitor, IκB, and translocates to the nucleus where it initiates the translation process of a pro-inflammatory cascade of cytokines and other mediators [21]. Decreased E-cadherin and increased vimentin levels are considered markers of EMT, a process by which a cell loses the characteristics of the epithelial phenotype while increasing its mobility and gaining the ability to migrate [22]. These data suggest the existence of an IGFBP2/p65/EMT axis in PDAC tumors. Both IHC staining and clinical data have confirmed that IGFBP2 promotes EMT by NF-κB, which may contribute to lymph node metastasis and poor prognosis for PDAC patients [20].

The mechanism of the role of IGFBP2 in PDAC development is complex and still unclear. The IGFBP2/p65/EMT axis may provide a potential direction for further research into PDAC advancement. It seems that IGBP2 may serve as a good marker in distinguishing CP patients from those with PDAC. Moreover, the ratio of IGF-1 to IGFBP2 is characterized by high sensitivity and specificity, which may be helpful in the detection of PDAC.

### 2.5. Lysophosphatidic Acid (LPA) and Autotaxin (ATX)

The lysophospholipid mediator lysophosphatidic acid (LPA) is a bioactive phospholipid that, by combining with the appropriate LPAR1-6 receptor, participates in cell migration, proliferation, and differentiation [23].

One study analyzed whether LPA receptors affect the invasive activity of PDAC cells. Two cell lines were used: high-invasion PANC-R9 cells and PANC-1 cells. The PANC-R9 cells had 15 times higher invasive activity than the PANC-1 cells. It was shown that LPAR1 expression was significantly elevated in PANC-R9 compared to PANC-1 cells, and LPAR3 expression was decreased. These results suggest that LPA signaling though LPAR1 could contribute to pancreatic cancer cell invasion [24].

Autotaxin (ATX) is a tumor-cell-motility-stimulating factor that was originally identified in melanoma cells. Apart from the phosphodiesterase function, it has lysophospholipase D (lysoPLD) activity that hydrolyzes lysophosphatidylcholine (LPC) to LPA and regulates LPA levels in serum [25].

Elevated ATX-LPA signaling activity is aberrantly expressed in many human cancers including breast and pancreatic cancer [26].

Stromal metabolic changes during PDAC progression, such as increased lipid production in pancreatic stellate cells (PSCs) that may nurture cancer tissues in a paracrine way, have been observed. An increased release of LPC from stromal cells was shown. The strong expression of autotaxin has been observed in tumors. In contrast, the inhibition of autotoxin expression by autotaxin inhibitor ONO-8430506 was found to reduce tumor growth by 2-fold [27].

Serum ATX activity was measured in patients with cancer of the esophagus (*n* = 8), stomach (*n* = 18), colorectum (*n* = 21), biliary tract (*n* = 19), and pancreas (*n* = 103), as well as in benign pancreatic diseases (*n* = 73). The ATX activity was only elevated in PDAC patients and not in other cancers, CP, or pancreatic cysts. The serum ability of ATX in the diagnosis of PDAC expressed by AUC was found to be 0.541 (95% CI: 0.435–0.648) for men and 0.772 (95% CI: 0.659–0.885) for women. At various cut-off points of serum ATX activity, the authors observed the high specificity but low sensitivity of the serum ATX activity for diagnosing PDAC. Further studies to evaluate the utility of elevated serum ATX activity in the diagnosis of PDAC are needed [28].

In 114 patients with PDAC, 94 subjects with benign pancreatic diseases (BPDs) such as benign biliary obstruction or chronic pancreatitis, and 120 healthy subjects, ATX, LPA, and Ca19-9 concentrations of were measured with ELISA. Those marker levels in PDAC patients were significantly higher than in healthy subjects (*p* < 0.001) and the BPD group (*p* < 0.001). The 10.7 µg/mL cut-off value for LPA had 91.74% sensitivity and 69.4% specificity for the differentiation of PDAC from healthy volunteers and benign pancreatic diseases. In early-stage PDAC, the LPA had a sensitivity of 80.67% and a specificity of 69.4%. The ATX cut-off of 286 ng/mL had a sensitivity of 78.95% and a specificity of 80% for the detection of PDAC among the entire study population, but this sensitivity decreased to 65.33% for the detection of early-stage PDAC. A combination of ATX, LPA, and Ca19-9 improved diagnostic accuracy for early-stage PDAC compared to a control group with an AUROC of 0.983 ± 0.016 (*p* = 0.0012). A panel of ATX, LPA, and Ca19-9 enhanced the AUROC to 0.973 ± 0.023 (*p* = 0.0090) compared to the BPD group [29]. The levels of all three biomarkers could be used to detect early-stage PDAC patients from patients with BPD or from healthy populations.

Serum ATX and LPA activity could have potential roles in identifying PDAC patients. It seems that more extensive research is needed to further evaluate the usefulness of those markers.

### 2.6. Multiple Biomarkers Panel

The field of cancer diagnostics is increasingly focusing on panel-combined biomarkers, because this approach yields improved sensitivity and specificity in combination with Ca19-9. The use of combinations of biomarkers results from the heterogeneity of the microenvironment and the tumor itself [5]. 

In a cohort study, a biomarker panel consisting of Ca19-9, leucine-rich glycoprotein alpha-2 (LRG1), and transthyretin (TTR) was developed with multiple reaction monitoring mass spectrometry (MRM-MS) and enzyme-linked immunosorbent assay (ELISA). LRG1 is a protein that directly binds to the TGF-β accessory receptor and results in the promotion of the pro-angiogenic Smad1/5/8 signaling pathway [30]. Elevated levels of protein have been found in the blood of patients with non-small cell lung cancer, colorectal cancer, and PDAC [31].

TTR is a functional protein in the pancreatic β-cell. It is involved in promoting insulin release and protecting against β-cell death. TTR can enter the pancreatic duct system due to hyperplasia and architectural destruction in PDAC tissues [32].

The resulting panel with three markers showed a sensitivity and specificity of 82.5% and 92.1%, respectively, to distinguish PDAC patients (*n* = 80) from healthy subjects (*n* = 89), and it increased the diagnostic performance of Ca19-9 alone by 10% in all stages of PDAC. The levels of Ca19-9 and LRG1 increased, and that of TTR declined. The use of this panel for the early detection of PDAC was assessed in stage I/II of PDAC compared (*n* = 50) to healthy subjects (*n* = 89). Ca19-9 had an AUC value of 0.792 (sensitivity = 64.0%), whereas the combined biomarkers improved the AUC value to 0.907–0.914 with a sensitivity of 76.0% and a specificity of 78.0%. The levels of Ca19-9 and LRG1 increased and the level of TTR decreased in early PDAC stages. The specificity of the panel was measured in 21 cases of benign pancreatic disease. Compared to Ca19-9 (AUC = 0.812), the panel discriminated PDAC from other pancreatic disease, such as IPMNs, with an AUC value of 0.895 (specificity = 85.7%; sensitivity = 82.5%) [33]. This lretrospective= study demonstrated the panel’s high diagnostic accuracy and ability to complement Ca19-9 in detecting early-stage PDAC.

In another study, a new panel of biomarkers—Ca19-9, apolipoprotein E (Apo-E), inter-alpha-trypsin heavy chain inhibitor H3 (ITIH3), apolipoprotein 1 (Apo-A1), and apolipoprotein L1 (Apo-L1)—showed 95% sensitivity and 94.1% specificity for the diagnosis of 40 PDAC patients compared to healthy subjects (*n* = 34) [34].

A panel consisting of TFF1, TFF2, and TFF3 (trefoil factors 1, 2, and 3, respectively) was evaluated in 80 patients with early-stage PDAC (I and II), 73 patients with advanced-stage PDAC (III and IV), and 47 patients with CP [35]. TFFs are stable mucin-associated proteins expressed in the gastrointestinal mucosa. There are known to protect the gastric mucosa from inflammation and accelerate epithelial injury. Their role as oncogenes has been observed in multiple malignancies, including colon, breast, prostate, and ovarian cancers [36].

A combination of all TFFs and Ca19-9 provided a sensitivity of 85% and a specificity of 92% for the detection of stage I and II PDAC. This panel combination showed better performance in differentiating CP from early-stage PDAC, with a sensitivity of 92% and a specificity of 90% compared to Ca19-9 alone [35].

A six-plex immunoassay comprising macrophage inhibitory cytokine-1 (MIC-1), carcinoembryonic antigen cell adhesion molecule-1 (CEACAM-1), osteopontin (OPN), melanoma inhibitory activity (MIA), spondin-1 (SPON1), and heat shock protein 27 (HSP27) demonstrated a potential advantage over Ca19–9 alone in the early detection of PDAC [37].

The MIC-1 protein belongs to the TGF-β family. MIC-1 has been shown to exhibit antitumorigenic and proapoptotic activities and to be overexpressed in breast, gastric, colorectal, and pancreatic cancers [38]. Serum ELISA MIC-1 values have been helpful in differentiating patients with resectable PDAC from healthy patients with an AUC of 0.99 compared to an AUC of 0.78 for Ca19-9 alone [39]. OPN is protein mostly expressed in bone. It has been shown that OPN promotes the production of inflammatory cytokines such as IL-17. OPN is related to tumor progression through binding to CD44 and integrin or promoting the invasiveness of cancer cells [40]. OPN was found to be expressed in PDAC, and its level of over 102 ng/dl allowed for the differentiation of PDAC from CP (*p* < 0.001) [41]. MIA is a protein that interacts with extracellular matrix proteins. The overexpression of MIA in melanoma cells promotes metastatic behavior. In PDAC tissues, the overexpression of MIA was found, but its serum levels could not detect a difference between healthy and PDAC patients [42]. CEACAM-1 is a member of the glycosylphosphatidylinositol (GPI)-linked immunoglobulin (Ig) superfamily that is expressed on the surface of endothelial, hematopoietic, and epithelial cells of different organs. CEACAM-1 was shown to be overexpressed in colon, breast, lung, and pancreatic cancer. CEACAM-1 plays a role in pro-angiogenetic effects, the regulation of the cell adhesion, and apoptosis [43]. It was reported that CEACAM-1 was expressed in serum of 91% (74/81) of studied PDAC patients, 24% (15/61) of control patients, and 66% (35/53) of CP patients, with sensitivity and specificity values greater than those of Ca19-9 alone [44]. This panel was evaluated in PDAC patients, IPMN patients, CP patients, and healthy controls. It was shown that two-marker panels of Ca19–9 and MIA significantly improved the differentiation of early-stage PDAC from CP with an AUC of 0.86. In addition, it was reported that Ca19-9 and MIC-1 together could distinguish early-stage PDAC from IPMN with an AUC of 0.81 [37]. A panel consisting of Ca19–9, MIC-1, and OPN was more useful than the individual biomarkers in the differentiation of IPMN from CP, with an AUC of 0.81. A three-marker panel of Ca19–9, CEACAM-1, and MIA differentiated patients with pancreatic cancer from benign pancreatic conditions such as CP, with an AUC of 0.86.

Accumulating data show that combinations of biomarkers are more effective and accurate than single biomarkers in the diagnosis of PDAC. Serum biomarker panels may reach sensitivity, specificity, and overall accuracy levels unlikely for a single biomarker such as Ca19-9. The combination of markers might improve early diagnosis, decrease screening costs and treatment, and prolong the survival of PDAC patients. Moreover, biomarker panels can improve the quality of life by determining prognosis and helping to avoid invasive procedures and ineffective treatment. Here, we present a comprehensive list of biomarkers used for the early diagnosis of PDAC patients (Table 1 ).

## 3. Prognostic Biomarkers

### 3.1. Ca19-9

A multicenter study included 274 PDAC patients who received neoadjuvant chemotherapy (NAC) with FOLFIRINOX (FLX) (5-flurouracil, folinic acid, oxaliplatin, and irinotecan) or gemcitabine/nab-paclitaxel (GNP) followed by curative-intentioned pancreatectomy. The authors analyzed clinical and biochemical response for NAC. The results showed that a biochemical response characterized by decrease level of Ca19-9 ≥50% was associated with better overall survival (OS), recurrence-free survival (RFS), and metastasis-free survival (MFS) rates compared to a Ca19-9 decrease level of <50% (OS: 42.3 vs. 24.3 months, *p* < 0.001; RFS: 27.3 vs. 14.1 months, *p* = 0.042; MFS: 29.3 vs. 13 months, *p* = 0.047). It was suggested that a greater decrease in Ca19-9 after treatment indicates better survival [45].

A retrospective analysis of 152 unresectable PDAC patients treated from 2007 to 2019 evaluated Ca19-9 as prognostically significant. It was shown that OS and PFS were shorter in PDAC patients with Ca19-9 ≥90 IU/mL than in a group with a Ca19-9 value of <90 IU/mL (PFS: 4.4 vs. 17.0 months, respectively, *p* < 0.001; OS 7.4 vs. 26.1 months, respectively, *p* < 0.001). The results confirmed a significant association between elevated Ca19-9 levels and reduced survival results in PDAC patients [46].

### 3.2. Neutrophils to Lymphocytes Ratio (NLR)

Chronic inflammation plays an essential role in carcinogenesis, including tumor initiation, promotion, malignancy, invasion, and metastasis induction [47].

A severe inflammatory response has been recognized as a poor prognostic indicator in various malignancies such as colorectal cancer, non-small cell lung cancer, and PDAC [48,49].

Therefore, inflammatory response markers such as the NLR (neutrophil to lymphocyte ratio) index and the modified GPS (Glasgow prognostic score) have been developed. The NLR is determined by blood count as the ratio of the absolute number of neutrophil granulocytes to the absolute number of lymphocytes. A normal NLR is roughly 1–3, and an NLR of 6–9 suggests mild inflammation, as seen in a patient with uncomplicated appendicitis [49]. 

The NLR is a marker of the systemic inflammatory response, and many studies have proven its value in predicting the prognosis of various cancers. The systemic inflammatory response to neoplasia is associated with neutrophil infiltration. Neutrophils secrete factors that stimulate tumor progression, such as interleukin-2 (IL-2), interleukin-6 (IL-6), interleukin-10 (IL-10), tumor necrosis factor α (TNF-α), and vascular endothelium growth factor (VEGF). Moreover, thesecretions of TNF-α and IL-10 lead to a reduced number of lymphocytes, and their dysfunction [50].

A meta-analysis that included 43 cohort studies evaluated the prognostic value of the NLR in patients with pancreatic cancer. The results showed that patients with low NLR values might have longer OS rates compared to patients with high NLR values (HR = 181; 95% CI = 1.59–2.05; *p* < 0.00001; I^2^ = 82%). Moreover, a low NLR was significantly associated with a longer disease-free survival (DFS) compared to a high NLR in PDAC (HR = 1.66; 95% CI = 1.17–2.35; *p* = 0.005; I^2^ = 67%). Patients with low NLR values had significantly smaller tumor sizes (*p* = 0.0007), better differentiation (*p* = 0.003), earlier stage diseases (*p* = 0.02), and low Ca19-9 levels (*p* = 0.007) than the high NLR patients. In the presented studies, the cut-off values of the NLR varied from 2.0 to 5.0. This meta-analysis did not provide the optimal cut-off value for the NLR in clinical practice. The accumulative results strongly support the idea that the NLR may be a promising prognostic marker for PDAC [51].

In a single-center cohort study, the association between NLR scores and PDAC prognosis in a group of 212 advanced-stage PDAC patients following palliative chemotherapy was evaluated. The results showed that pretreatment for those with an NLR ≥ 5 was correlated with shorter survival times compared to patients with an NLR < 5 (6 vs. 12.8 months, respectively). It was suggested that an elevated NLR ≥ 5 might be a promising biomarker to distinguish PDAC patients with poorer prognosis following palliative chemotherapy [52].

In another study, the inflammatory-based scores of patients with advanced pancreatic PDAC who received first-line chemotherapy were assessed. A post-treatment NLR value of more than 2.62 was associated with poor OS and recurrence-free survival (RFS) rates compared to patients with an NLR score of lower than 2.62 (OS 3.9 months vs. 11.0 months, respectively, *p* < 0.001; PFS 2.0 months vs. 3.6 months, respectively, *p* < 0.001). The authors suggested that the NLR could be used as a prognostic biomarker and an adjunctive tool for assessing the response to chemotherapy in advanced-stage PDAC [53].

It was shown that a panel consisting of the NLR and Ca19-9 had significant prognostic value for OS following PDAC resection. The preoperative parameters of an NLR of higher than 2.7 and a Ca19-9 level of higher than 230 IU/mL were correlated with poor survival. Additionally, the same values of the NLR and Ca19-9 were connected to a 37.5% overall 2-year survival rate compared to an 89.9% overall 2-year survival rate in patients with lower levels of the NLR and Ca19-9 [54].

An increasing number of scientific papers are suggesting that neutrophil infiltration around a tumor is associated with poor survival, while decreased lymphocyte counts result in an inadequate immune response to cancer cells, thus resulting in a poorer prognosis. The ratio of elevated neutrophils and decreased lymphocytes could be most valuable in predicting prognosis [53]. A high NLR was found to be strongly correlated with poor prognosis and shorter OS in PDAC patients. Accumulated evidence has demonstrated heterogeneous NLR cut-off values, so future large-scale studies are needed to confirm the optimal cut-off point of the NLR.

### 3.3. mGPS

The mGPS index is based on serum CRP and albumin levels. Inflammatory cytokines (notably interleukins IL-1, IL-6, and TNFα) released from tumor cells induce CRP protein production in hepatocytes. Albumins are negative acute phase proteins synthesized in the liver, and their value decreases in response to inflammation. In addition, albumin had numerous biologic functions, such as maintaining osmotic pressure, transporting metabolites, and antioxidant activity. Low albuminemia was found to reflect malnutrition in patients including those with different malignancies [55]. A low preoperative serum albumin level was found to be correlated with poor OS and RFS and increased morbidity and mortality in patients with urothelial carcinoma [27]. Additionally in patients with non-small cell lung cancer (NSCLC), low preoperative albumin values were shown to be an unfavorable independent prognostic factor for DFS (*p* = 0.001) and OS (*p* = 0.001) [28]. It was suggested that mGPS allows for the better assessment of systemic inflammation or malnutrition with both albumin and CRP changes [56,57].

Recent studies have suggested a high mGPS index value as a prognostic factor. Patients with a CRP level of ≤10 mg/dl and an albumin level of ≥35 g/L or <35 g/L were assigned a score of 0, patients with increases of both CRP (>10 mg/L) and hypoalbuminemia (<35 g/L) were assigned a score of 2, and patients with an increase of CRP only were assigned a score of 1 [58]. 

A meta-analysis including 6512 patients evaluated the prognostic value of the mGPS in patients with PDAC. mGPS levels of 1 or 2 were significantly more often associated with poor OS compared to an mGPS level of 0 (mGPS = 1 vs. mGPS = 0, HR = 1.68, 95% CI: 1.25–2.27, *p* = 0.001; mGPS = 2 vs. mGPS = 0, HR = 1.90, 95% CI: 1.36–2.67, *p* < 0.0001). Additional further analyses of 12 studies indicated that mGPS values of 1 or 2 correlated with worse OS in patients receiving chemotherapy (HR = 1.45; 95% CI: 1.05–2.02; *p* = 0.025) and after pancreatectomy (HR = 1.64; 95% CI: 1.25–2.15; *p* < 0.0001) [59]. 

In 72 patients with inoperable PDAC treated with palliative chemotherapy, the mGPS index and NLR proved to be the most reliable for prognostic evaluation among other inflammatory and nutritional markers including the platelet-to-lymphocyte ratio (PLR), the prognostic nutritional index (PNI), and a controlled nutritional status score (CONUT). The PLR is the ratio of the peripheral absolute platelet count divided by the absolute lymphocyte count. The PNI is an objective assessment index reflecting the immune–nutritional status of patients, and it is calculated via the multiplication of the serum albumin level and the lymphocyte count. The CONUT score is an index calculated from the serum albumin concentration, the total peripheral lymphocyte count, and the total cholesterol concentration. The CONUT score is a screening tool used to identify undernourished patients. Among them, an NLR ≥ 4 (*p* < 0.001) and an mGPS of about 2 (*p* = 0.005) were found to be independent prognostic factors for PFS in multivariate analysis. The median OS in patients with elevated mGPS levels of about 2 and an NLR of more than 4 was found to be 97 days; in contrast, patients with lower values of those markers showed a median OS of 342 days) The PFS time of patients with an mGPS score of 2 was significantly poorer than that of patients with mGPS scores of 0 and 1 according to a Kaplan–Meier analysis (*p* < 0.001). The mGPS was found to be most useful inflammatory marker to predict prognosis in unresectable PDAC patients treated with chemotherapy [55].

In conclusion, mGPS might be a novel and promising inflammatory prognostic biomarker in PDAC patients. mGPS is a widespread biomarker available in everyday clinical practice, and its values can divide patients with advanced PDAC into groups with different survival probabilities, thus allowing for the selection of targeted treatment and appropriate clinical decisions.

### 3.4. Factors of Coagulation

The hyperactivation of the coagulation system and secondary increased fibrinolytic activity are found in many patients with malignant tumors. Patients with PDAC are predisposed to developing venous thromboembolic events (VTEs), which predict poor prognosis. In PDAC, significantly elevated plasma levels of fibrinogen and D-dimer have been observed [60]. 

D-dimers are fibrin-degradation products, and their increase may indicate a hypercoagulable state and secondary increased fibrinolytic activity [61].

Fibrinogen (FBG) is a soluble glycoprotein that is normally synthesized by the liver and released into the blood. In addition, it participates in the systemic inflammatory response as an acute inflammatory protein. In the coagulation process, soluble fibrinogen develops into insoluble fibrin and ultimately forms a blood clot [62]. 

A retrospective study investigated the impact of preoperative plasma D-dimer levels in predicting the survival of PDAC patients undergoing radical resection. In 417 of 1351 PDAC patients, higher preoperative plasma D-dimer levels (≥0.55 mg/mL) were observed. Patients with elevated preoperative D-dimer levels had significantly shorter OS than those with low D-dimer levels (15.0 months vs. 21.3 months, respectively; *p* < 0.001). The results showed that D-dimer was a reliable prognostic factor in patients who underwent R0 resection (*p* < 0.001). Moreover, an analysis of preoperative D-dimer, Ca19-9, and NLR values in PDAC patients receiving radical surgery enhanced the prognostic accuracy for OS [63]. The prognostication of patients with PDAC who undergo surgery might be predicted by assessing the D-dimer level as a promising and reliable marker.

A recent study examined the prognostic significance of FBG combined with D-dimer in PDAC patients undergoing radical R0 resections. A cohort of 282 patients with PDAC was included. Patients were further divided into high and low preoperative FBG and D-dimer values. The cut-off values were 3.31 g/L for FBG and 0.53 mg/L for D-dimer. They found that patients in the low-concentration group had a longer median OS of 31.17 months than those in the high-concentration group with a median OS of 15.43 months (high group vs. low group, HR: 2.397, 95% CI: 1.723–3.335; *p* < 0.001). Therefore, low preoperative FBG and D-dimer concentrations may indicate better prognosis in PDAC patients undergoing radical R0 resection. The authors suggested that the reduction of fibrinogen and D-dimer levels with anticoagulant therapy may have a positive effect on the prognosis of PDAC patients undergoing R0 resection [64].

A recent study of 320 patients diagnosed with advanced PDAC evaluated the prognostic significance of hemostatic parameters such as prothrombin time (PT), activated partial thromboplastin time (APTT), FBG, platelet count (PLT), mean platelet volume (MPV), plateletcrit (PCT), and platelet distribution width (PDW). The results showed that several hemostatic parameters, including PT > 11.3 s (HR = 1.43; 95% CI = 1.08–1.90; *p* = 0.014), FBG > 2.5 g/L (HR = 1.39; 95% CI = 1.06–1.81; *p* = 0.016), and MPV > 12.2 fL (HR = 1.42; 95% CI = 1.06–1.90; *p* = 0.020), were independent prognostic factors for poor OS. Moreover, all the patients were divided into three groups according to scoring system based on those markers. A novel score was calculated by the following formula: 1 point for high PT (>1.3), high FBG (>2.5), or high MPV (>12.2) and 0 points for low PT (≤11.3), low FBG (≤2.5), or low MPV (≤12.2). The low-risk group was assigned a score of 0 or 1 (*n* = 106; 33.1%), the median-risk group was assigned a score of 2 (*n* = 180; 56.5%), and the high-risk group was assigned a score of 3 (*n* = 34; 10.6%). The median survival time of patients in the low-risk, median-risk, and high-risk groups were 8.8 months (95% CI = 6.8–10.9), 6.3 months (95% CI = 5.3–7.3), and 4.3 months (95% CI = 2.6–5.9), respectively (*p* < 0.001). The scoring system showed prognostic value in both III (*p* < 0.001) and IV (*p* = 0.036) stage PDAC TNM. In addition, in the high-risk group, stage III patients had even shorter survival times (about 4.2 months) compared to the stage IV patients (about 5.3 months). The combination of PLT, FGB, and MPV was found to be an independent prognostic factor for OS in advanced PDAC patients. The panel of indicators showed prognostic value in both stage III and IV PDAC patients. The authors suggested that more attention should be paid to locally advanced high-risk patients (stage III TNM) who may have poorer prognosis than metastatic PDAC (stage IV TNM) [65]. Furthermore, large-scale and multicenter studies of early-stage PDAC should be conducted to investigate the relationship between the hemostatic system and PDAC.

There is ample evidence assessing hemostatic parameters regarding the survival of patients with PDAC. Pancreatic cancer significantly increases the risk of venous thromboembolism, which is associated with an increased mortality during hospitalization. In the future after extended studies, factors of coagulation may prove their utility in prognostics, the prediction of the incidence of thromboembolic events, and targeted treatment in advanced pancreatic cancer.

The overall survival of patients with PDAC has not been found to improve, even with the use of new diagnostic and therapeutic strategies. In addition to well-known prognostic factors, such as tumor stage, surgical margin, and Ca19-9 value, new prognostic biomarkers have been recently proposed. The promising prognostic value of the NLR has been shown. Prognostic factors can help guide personalized treatment in PDAC patients. The results of selected studies associated with prognostic biomarkers are listed in Table 2.

## 4. Predictive Biomarker

As a predicting marker, Ca19-9 can assess resectability, therapy efficacy, tumor recurrence, and long-term survival. One scientific report proposed the use of Ca19-9 to detect PDAC recurrence while bypassing imaging techniques, since CT or MR have their limitations in detecting small or diffuse foci located, for instance, in the peritoneum, which might lead to delays in starting relevant treatment [66].

In order to detect PDC recurrence after surgery, ESMO guidelines recommend monitoring Ca19-9 levels every 3 months for 2 years in patients with high preoperative Ca19-9 levels, along with abdominal computed tomography conducted every 6 months [67].

Subsequent authors reported that the observation of Ca19-9 level safter pancreatectomy predicts markedly worse RFS and may help indicate the direction of rescue chemotherapy. The study included 525 patients undergoing surgery for PDAC. The level of Ca19-9 was marked at diagnosis, after surgery, and at 6-month intervals. The patients were divided into distinct patterns of Ca19-9 behavior after resection with different involvements in RFS and OS. The patients identified in the post-resection group had a persistently normal level of Ca19-9 (18.7%), which was connected to a lowest RFS; the period of normal marker values was followed by an increase (4.9%) that was characterized by a worse RFS and short OS; and the last period, with elevation and decline during follow-up (23.6%), was not associated with the risk of recurrence or death. Additionally, the authors compared the increase of Ca19-9 at 6-month intervals and radiographic findings. The results showed that for radiographic recurrence, elevated levels of Ca19-9 had poor positive predictive value (average: 35%) but the normalization of Ca19-9 had high negative predictive value (average: 92%). The authors assumed that normal Ca19-9 levels indicate no or very low risk of recurrence on imaging, but elevated Ca19-9 levels are frequently inconsistent with recurrence in imaging techniques [68].

Moreover, the connection between increases in Ca19-9 and radiological recurrence was investigated in a follow-up study after PDAC resection. Ca19-9 was measured at diagnosis, after surgery, after adjuvant chemotherapy, and at a validation point in 134 patients. The optimal cut-off point was the ratio of the postoperative Ca19-9 value (before image recognition for relapse patients) to the first available postoperative Ca19-9 value. The Ca19-9 values and CT and MR findings were compared. The median follow-up time was 644 days (22 months). The authors observed that approximately 60% of the patients after resection showed significantly elevated Ca19-9 values before the detection of recurrence in imaging techniques. Additionally, in the validation set, a 2.45-times elevated Ca19-9 level was found to indicate recurrence with a sensitivity of 90% and a specificity of 83.33%, with an AUC of 95%. Concomitant Ca19-9 elevation and CT recurrence detection was found only in four (22.2%) patients. The median progression-free survival (PFS) of the patients with Ca19-9 elevation during first-line palliative chemotherapy prior to the imaging detection of cancer recurrence was 97 days (95% CI: 79–128), while in patients with no prior Ca19-9 elevation, it was 280 days (95% CI: 167–280). This may have been the result of delayed palliative chemotherapy. The authors suggested that Ca19-9 monitoring after PDAC resection could be helpful in deciding on patients’ management even without imaging results. Close Ca19-9 observation could detect PDAC recurrence or metastases, even several months before their clinical or radiologic evidence. Therefore, these data suggest that the routine monitoring of Ca19-9 levels may improve patient outcomes and survival [69].

Recently, the results of a retrospective analysis regarding the usefulness of multiple assessments of Ca19-9 levels after NAC and subsequent resection were published. It was concluded that a Ca19-9 level of ≤103 IU/mL, tumor size of ≤27 mm, and the lack of lymph node metastasis and R0 resection were significant predictors of survival benefits. Moreover, PDAC patients with elevated Ca19-9 levels of >37 IU/mL before NAC and with decreased levels of ≤37 IU/mL afterwards were associated with a lower risk of hepatic recurrence (18%) compared to patients with Ca19-9 values of >37 IU/mL after NAC (31%). The authors suggested that Ca19-9 monitoring could be used to assess the efficiency of NAC therapy [70].

It was reported that 40% of patients with borderline resectable (BRPC) and locally advanced pancreatic cancer (LAPC) cannot achieve surgical resection after NAC. The decision about which patients are qualified for potential resection following NAC is clinically difficult, so whether Ca19-9 levels during NAC could predict tumor resectability was investigated. Ca19-9 levels were analyzed at diagnosis and before and after FLX or GNP NAC in patients with BRPC and LAPC. The study showed that a decrease in the Ca19-9 concentration to a value lower than 91.8 IU/mL after FLX NAC could independently predict tumor resectability in patients with BRPC or LAPC. For patients with a cut-off above 91.8 IU/mL, resection was impossible due to distant metastases. In the GNP group, no relevant cut-off values could be identified. The authors suggested that a decrease in Ca19-9 levels after NAC may predict tumor resectability and should be included as one of the qualifying factors for surgical decision making [66].

Elevated postoperative Ca19-9 levels were found to be correlated with worse prognosis and hepatic recurrence after surgery. A recent study enrolled 539 consecutive patients with PDAC who underwent R0 resection; there were two group patients, first with sustained high levels of Ca19-9 after surgery and second with no postoperative elevation of Ca19-9. It was shown that postoperative sustained elevation was associated with a shorter median OS compared to patients with normal levels of Ca19-9 (17.1 vs. 35.4 months, respectively; *p* < 0.0001). Postoperative Ca19-9 elevation was 2.6 times more consistent with hepatic recurrence than in the group without elevation (45% vs. 17%, respectively; *p* < 0.0001) [71]. Postoperative Ca19-9 elevation after resection is a strong independent predictor for survival and could indicate the presence of occult distant metastasis in patients with PDAC. Furthermore, patients with increased postoperative Ca19-9 levels may require intensive adjuvant therapy. 

In conclusion, Ca19-9 serum measurement is becoming more useful in assessing treatment efficacy and predicting outcome, recurrence, and response to chemotherapy than in the early diagnosis of PDAC. The routine monitoring of Ca19-9 levels after NAC could detect PDAC recurrence or metastases before their clinical or radiologic evidence.

## 5. Conclusions

In recent years, many studies identifying novel PDAC biomarkers and biomarker panels have emerged. Nevertheless, none of these biomarkers have been established in clinical practice. These biomarker studies have been based on relatively low case numbers and lack validation.

Accumulating data have shown that the combinations of biomarkers are more effective and accurate than single biomarkers in the diagnosis of PDAC. Serum biomarker panels may reach high levels of sensitivity, specificity, and overall accuracy unlike single biomarkers. Moreover, combinations of biomarkers have shown better diagnostic accuracy and the ability to complement Ca19-9 in detecting early-stage PDAC.

Further understanding the complexities of cancer biology can help develop clinically useful markers for the early detection and prognosis of PDAC. Large prospective studies are needed to investigate the clinical impact of including these biomarkers in clinical decision making to improve outcomes for this disease. The discovery of accurate biomarkers may also allow for the better stratification of patients and guide therapeutic choices.

## Figures and Tables

**Table 1:** Biomarkers used for the early diagnosis of PDAC patients.

Diagnostic Biomarkers	Examined Population	Sensitivity [%]	Specificity [%]	AUC	PPV [%]	Ref.
Ca19-9	Symptomatic patients with suspected PDAC	79–81	82–90	NA	NA	[1]
Ca19-9	Healthily population	100	98.5	NA	0.5–0.9	[7]
Ca19-9 and THBS-2	All stages of PDAC I/II stage of PDAC III/IV stage of PDAC	90 98 NA	90 87 NA	0.952 0.960–0.971 0.911–0.980	NA NA NA	[11,12]
IGF-1/IGFBP2 ratio	All stages of PDAC	100	97	NA	NA	[18]
Ca19-9, ATX, and LPA	I/II stage of PDAC	NA	NA	0.983	NA	[29]
Ca19-9, LRG1, and TTR	All stages of PDAC	82.5	92.1	0.931	NA	[33]
CA19-9, Apo-E, ITIH3, Apo-A1, and Apo-L1	All stages of PDAC	95	94.1	0.99	NA	[34]
Ca19-9, TFF1, TFF2, and TFF3	I/II stage of PDAC	90	92	0.93	NA	[35]
Ca19–9, MIC-1, and OPN	All stages of PDAC	NA	NA	0.99	NA	[37]

**Table 2:** Survival and recurrence-free survival outcome of PDAC patients.

Prognostic Factor	Subject/Treatment	Outcome	Overall Survival	RFS or PFS	Reference
Ca19-9	NAC followed by resection	Decrease <50% vs. ≥50% after NAC	24.3 vs. 42.3 months	RFS: 27.3 vs. 14.1 months	[45]
Ca19-9	Unresectable PDAC	≥90 IU/mL vs. <90 IU/mL	7.4 vs.26.1 months	PFS: 4.4 vs. 17.0 months	[46]
NLR	NAC	>5 vs. <5 after NAC	6 vs. 12.8 months	NA	[52]
NLR	Unresectable PDAC	>2.62 vs. <2.62	3.9 vs. 11.0 months	PFS: 2.0 vs. 3.6 months	[53]
NLR and mGPS	Unresectable PDAC	NLR > 4 and mGPS = 2 vs. NLR < 4 and mGPS < 2	3.2 vs. 11.4 months	NA	[55]
D-Dimer	R0 resection	Preoperative ≥ 0.55 ng/mL vs. < 0.55 ng/mL	15.0 vs. 21.3 months	NA	[63]
D-Dimer and FBG	R0 resection	Preoperative FBG > 3.31 and D-dimer > 0.53 mg/L vs. FBG < 3.31 g/L and D-dimer < 0.53 mg/L	15.43 vs. 31.17 months	NA	[64]

## Data Availability

The data presented in this study are available on request from the corresponding author.
